# Some Cape Town Institutions

**Published:** 1897-07-10

**Authors:** 


### SOME CAPE TOWN INSTITUTIONS.

The first view of Cape Town—that obtained from the steamer after entering Table Bay—is undoubtedly the best to be obtained anywhere, if we except the view from Robben Island, which is practically the same. The town is literally embraced by Table Mountain, which forms a huge semi-circle above and around it, the utmost spurs running out to the sea. Once, long ago, the whole of this vast enclosure was a forest of the beautiful silver fir, groves of which still clothe the slopes of the mountain, but in greatly diminished numbers; it is said to be by degrees becoming extinct which would be a pity, indeed, for this is the only place in the world where it is known to grow.

On entering the docks a high hill is seen on the left hand, crowned by a large, fine-looking white building. This is the New Somerset Hospital, probably the best hospital, excepting, of course, Kimberley, in the Colony; its commanding position, with wide views towards the mountain on one side, and the bay on the other, would alone make it a cheerful and attractive place. The inside corresponds to the outside, and well repays the exertion of a rather long and steep walk, although an omnibus also passes the door. Its name distinguishes it from the Old Somerset Hospital, which is situated quite in the town, with no advantages of position or construction. This was formerly the only lunatic asylum in Cape Town, but is now chiefly used as a chronic sick hospital, a last refuge for the old and helpless, the imbecile, and the incurable, among the poorer classes of white people and the natives.

Cape Town is, in some ways, very progressive; for instance, its principal streets are traversed in every direction by electric trams, which take you "all the way there" for threepence—a coin which always goes by the name of a "tickie" in the colony—and the course of which is marked by a perfect network of overhead wires. A local railway conveys one out of the town into the most lovely suburbs that ever a town possessed (with apologies to Tooting and Norwood). These suburbs are situated at the back of Table Mountain, but the railway continues all round the shores of False Bay to Simons Town, which is the terminus. Most of the inhabitants of Cape Town have their private residences in one or other of the suburbs, the town itself being hot, insanitary, and odorous. Sanitation, by the way, is in a very elementary stage all over the Cape Colony, in consequence of which there are continual epidemics of typhoid in all the towns. This must be in some measure ascribed to the preponderance of Dutch members in Parliament, who think sanitation quite superfluous, and are averse to wasting the public money on such absurd trifles as waterworks, bath-houses, drainage, and so forth. They argue that they are quite as healthy, and live quite as long, without baths as with them, and it cannot be denied that the facts seem to support them, so far. Moreover, they consider that English people must be very dirty to require washing every day, and, of course, there is something to be said for this point of view! But this is a digression.

Rondebosch is one of the prettiest of these suburbs, and here is the residence of Mr. Rhodes, Grootschuur, with its extensive grounds, all open to the public. Here also there has sprung up recently a new institution, a large and imposing building, erected by a sect or community who call themselves Seventh Day Adventists. What their peculiar tenets are beyond that, like the Jews, they keep Saturday instead of Sunday, I have had no opportunity of learning, but I believe that Mr. Selous came upon them as far up as Matabeleland.

Some of their views on other subjects besides religion seem to be peculiar. To give a minor instance, they call their institution (which appears to be a sort of convalescent home) a Sanatarium; a pleasing variation from the old-fashioned and hackneyed term Sanatorium. Moreover, they are very strict vegetarians; I believe that their diet sheet excludes even eggs and milk. This would seem to be a sort of starvation cure, suited for patients with apoplectic tendencies, but I am informed that a saving clause in the regulations permits the friends of the patients to bring them anything they like in the way of provisions—even a chicken! Personally, I should not care to recruit my health in that sanatorium—I should say sanatarium—unless I had a good many friends who were likely to think of me.

Before reaching Rondebosch another station is passed, named Observatory Road, because the Observatory is situated here. From this station, a long and very pretty, shady, private road leads up the hill to the new asylum at Valkenberg. It is difficult to say much about this building while it is still in its very unfinished, condition; but to judge from what is already completed, it will be really a very fine edifice some day. Of its magnificent position I have spoken in a previous letter; and besides the lovely view which it commands on all sides, its height above the sea gives it also the advantage that in the hottest days of summer there is almost always up here a cool breeze.

The old buildings of the asylum, which are small and inconvenient, are still in use; the male block, consisting of a large square court with the various buildings round it, is complete, but some part of it is now occupied by female patients, pending the completion of their own block. The administrative block is still in the hands of the carpenters and painters; but the front, with the main entrance, which can be seen from a distance, is very imposing. The building is two-storied, most of the dormitories being upstairs; all the wards and corridors are spacious, cheerful, and well lighted, those set apart for acute cases being as bright and pleasant as any. The hospital ward is particularly pretty and attractive, ending in a large bow window; the bath-rooms and lavatories are spacious and convenient, with the one exception that the hot water is not laid on, but has to be heated in boilers. The kitchen is very large, light, and airy, with every necessary appliance, a splendid range, and supplementary stoves; and there is a beautiful dining-hall, large and lofty, with a raised platform at one end for entertainments.

The accommodation for nurses is at present unsatisfactory, but it is hardly fair to speak of this, which is only owing to the unfinished state of the whole building. At present, also, the scattering of the patients, and the impossibility of making convenient and methodical arrangements, adds greatly to the work; but everyone who has lived in any place that is in course of building understands that many little discomforts and inconveniences must be cheerfully put up with during the transition stage, and the nurses at Valkenberg look forward to the time when their comfort will be fully provided for, and their labour lessened, by the satisfactory completion of every part of the building and all the internal arrangements thereof. To English ideas this progress is slow, and the transition stage unduly prolonged; but in the Colony things do not go on so rapidly as at home, everybody is less energetic, and here, again, the Dutch members of Parliament are in some degree responsible, in that Parliament has not yet granted the sum necessary to complete a work so well worthy of completion, and so valuable to the community.

